# An analysis of e-cigarette and polysubstance use patterns of adolescents in Bangkok, Thailand

**DOI:** 10.18332/tid/142894

**Published:** 2021-11-11

**Authors:** Bang-on Thepthien, Chit Su Tinn, Takuma Ofuchi, Bee Kim

**Affiliations:** 1 ASEAN Institute for Health Development, Mahidol University, Nakhon Pathom, Thailand; 2 Nuffield Department of Medicine, Trinity College, University of Oxford, Oxford, United Kingdom; 3 School of Social Sciences, London Metropolitan University, London, United Kingdom; 4 Addiction Science Department, SahmYook University, Namyang ju Si, South Korea

**Keywords:** e-cigarette, polysubstance use, adolescents, Thailand

## Abstract

**INTRODUCTION:**

The prevalence of adolescent e-cigarette use has increased markedly in recent years. Specifically, the prevalence of e-cigarette use over the past 30 days was higher than the prevalence of use of other tobacco products. However, there is no definitive data on e-cigarette use among adolescents, including a description of how e-cigarette use is part of a more widespread pattern of substance abuse. The objective of this study was to assess the prevalence of e-cigarette use in combination with tobacco, alcohol, or marijuana, and the risk of polysubstance use among a sample of Thai adolescents, analyzed by sociodemographic characteristics.

**METHODS:**

Data were extracted from the Bangkok Behavioral Surveillance Survey (BBSS) cross-sectional survey conducted in 2019. The survey used self-reports from a sample of adolescents aged 14–17 years in Bangkok (n=6167). Multinomial logistic regression was used to determine the status of poly drug use in combination with e-cigarettes.

**RESULTS:**

In all, 6.8% of adolescents in this sample reported having used e-cigarettes in the last 30 days. Among the students who used e-cigarettes, the majority (72.0%) reported using other substances along with e-cigarettes, and alcohol was the most common addictive substance used in combination with e-cigarette use. The use of e-cigarettes only and e-cigarettes in combination with other addictive substances (compared to the non-e-cigarette group) tended to be higher among male students, having low academic achievement, having a friend who smokes, being persuaded by a close friend, having ever had sex (OR: 1.48–3.70), and having close friends who drink alcohol (vs none) (OR=3.26).

**CONCLUSIONS:**

Polysubstance use is highly prevalent among adolescents who use e-cigarettes. There should be extensive screening for e-cigarette consumption, including use of other addictive substances, especially alcohol. Early and comprehensive prevention efforts to reduce the use of e-cigarettes and other addictive substances can have a huge impact on the health of the adolescent population.

## INTRODUCTION

In less than ten years since e-cigarettes were introduced, e-cigarettes have become the most commonly used tobacco product among adolescents in many countries, including Thailand which is experiencing increased use of e-cigarettes among youth. This is despite the fact that Thailand has prohibited the import and sale of e-cigarettes since 2014. A 2015 national survey of e-cigarette use among Thai middle school students found that 3.3% (4.7% of boys and 1.9% of girls) were current (past 30-day) users^1^. The overall prevalence of e-cigarette use in 2019 among students in grades 7–12 was 3.7% (5.9% for male, and 1.3% for female students); among youth aged 14–16 years, the prevalence of e-cigarette use was 6.4%^2^. There has been a significant increase in e-cigarette use among US youth since 2011^3^. A number of factors have contributed to the increase in e-cigarette use, including flavors that attract youth, and innovative delivery mechanisms and accessories^4,5^. This has led to the widespread use of e-cigarettes and the popularity of the product among US youth. The US Food and Drug Administration has implemented a policy that emphasizes the control of production, distribution, marketing, and selling of unlicensed flavored e-cigarettes^6^. A 2020 study in the US found that, in the past 30 days, 38.9% of high school students and 20.0% of middle school students had consumed e-cigarettes^7,3^. Among current users, more than 8 in 10 reported using e-cigarettes that contain flavoring and flavoring agents. Although the use of fruit-flavored e-cigarettes was common among e-cigarette users in 2020, the findings from studies in the US also highlight the prominence of menthol e-cigarette use, which accounted for nearly half of flavored, pre-filled pod or cartridge users and a quarter of flavored disposable product users^8,9^.

In Thailand, e-cigarettes are regulated as tobacco products. The current Tobacco Products Control Act bans selling electronic cigarettes or liquid for filling electronic cigarettes including renting, leasing, or providing for any reason whatsoever, including any offer or solicitation thereof. Any person who violates this law is liable for imprisonment for a term not exceeding five years and/or a fine not exceeding 0.5 million THB (1000 Thai Baht about 30 US$). According to the Consumer Protection Board Order No. 9/2015 dated 28 January 2015, the 2013 Consumer Protection Act (No. 3), which came into force on 18 February 2015, classifies ‘electronic cigarettes’ as a prohibited product, and it is unlawful to import e-cigarettes into Thailand. Any person violating this shall be liable to imprisonment for a term not exceeding ten years and/or a fine of five times the amount of the goods exported or imported (including forfeiture of the goods, the material used for packing the contraband, and the vehicle used to transport the products). The 2014 announcement of the Ministry of Commerce designated e-cigarettes as products prohibited to be imported into Thailand. However, at present, e-cigarettes are procured and shared among Thai teenagers, and these products are sold in/around entertainment venues, and traded directly on the Internet.

Products are advertised as having a good aroma, coming from non-toxic dried fruit, absent of nicotine, non-addictive, and can help tobacco addicts to quit smoking^10^. However, there is no reliable research with sufficient sample size of sufficient duration to conclude that e-cigarettes are effective in aiding cessation of traditional cigarette smoking^11,12^. The World Health Organization concluded that e-cigarettes did not reduce smoking of conventional tobacco products^13^.

Given the relative newness of e-cigarettes, there is a limited body of literature detailing the role that e-cigarettes play in alcohol consumption, cannabis use, and smoking tobacco products. Polysubstance use, particularly the practice of cannabis and alcohol co-use, is established in the literature, and referred to by the term ‘cross-fading’^14^ and this phenomenon has been extended to describe e-cigarette and alcohol co-use^15^. There are a few Thai research studies that have examined the combined use of e-cigarettes with other addictive substances among youth. However, those studies were conducted with a very small number of adolescents, and there was no analysis of the combined use of e-cigarettes with other addictive substances in Thai adolescents. Adolescents who reported using any nicotine products were more likely to report use of alcohol, marijuana, and other addictive substances^16^. Polysubstance use among smokers was significantly more prevalent compared to non-smokers^17^. Many of the polysubstance users also exhibited symptoms of substance-use disorder. There are too few studies on the association between use of multiple nicotine products and consumption of alcohol and other drugs^18,19^. Also, only a few studies have documented a positive relationship between e-cigarette and cannabis use. Furthermore, there are no studies on adolescent e-cigarettes being part of a more widespread pattern of substance abuse. Prior research shows that youth e-cigarette use is associated with residence in urban areas^20^. Therefore, the framework for a risk analysis of the current use of e-cigarettes in combination with other addictive substances has been developed to assess adolescent drug use among students in Thai public middle/high schools and vocational schools.

## METHODS

### Sample

Data come from the Bangkok Behavioral Surveillance Survey (BBSS) which, since 2002, has used self-administered questionnaires in the classroom setting, among representative samples of students in Bangkok. This secondary analysis examined data from the 2019 round of the BBSS. The BBSS used a multi-stage stratified sampling procedure to obtain a representative sample of public-school grades 8 and 11, and public vocational year II students. The first stage was selection of a geographical area that consisted of six primary areas. The second stage consisted of sampling of one or more schools within each of these six areas, with probability proportional to size. The third and final stage was the selection of students. Up to about 150 students of the target grade in the selected school were included in the data collection. In schools with fewer than 1000 students of the target grade, the usual procedure was to include the entire class in the data collection, when feasible. Student response rates in the 8th and 11th grades and vocational year II schools were 98%, 97%, and 95%, respectively; almost all non-responses were due to absenteeism. The sample for the 2019 BBSS included 6167 individuals. We restricted this analysis to adolescents who had provided complete data on their e-cigarette, alcohol, cannabis, and tobacco use.

The research protocol was approved by the Mahidol University ethical review committee. Consistent with school board requirements, parents provided permission for their child to participate in the study via active parent permission or active information/passive permission protocols. Only students with parental permission were invited to participate in the study on the day of survey implementation. Students were not remunerated and could withdraw at any time. We restricted the sample for the present study to those in high school.

### Measures

#### Dependent variable

The dependent variable was past 30-day e-cigarette poly-use status, coded as no e-cigarette use (reference), e-cigarette only use (single use), or e-cigarette + other substance use (poly use). We created a variable for frequency of e-cigarette use, in accordance with prior studies^6^ and coded as follows: none (0), infrequent (1–2 days), light (3–9 days), moderate (10–29 days), or daily (30 days).

#### Independent variables

The BBSS survey asked adolescents to report history of alcohol, cannabis, and/or tobacco use in the past 30 days. Cigarette use in the past 30 days was coded as 1 for respondents who reported any cigarette use in response to the question ‘How frequently have you smoked cigarettes during the past 30 days?’ and 0 for non-use. Marijuana use in the past 30 days was coded as 1 for respondents who reported any marijuana use in response to the question ‘On how many days did you consumed marijuana (weed, pot) during the last 30 days?’ and 0 for non-use. Alcohol use in the past 30 days was coded as 1 for respondents who reported alcohol use in response to the question ‘During the past 30 days, on how many days did you have at least one drink of alcohol?’ and 0 for non-use.

The BBSS assessed academic achievement with the following question: ‘During the past 12 months, how would you describe your grades in school?’. Response options were at an A, B, C or D level. We created a dichotomous indicator of academic achievement, coded as 0 for A + B (reference) versus C + D. For respondent’s report of having a close friend who uses one or more substances, the ‘close friend substance use’ scale consisted of three items which were adapted to capture close friends’ self-reports of drug use in the past 12 months. The three items include use of alcohol, cigarettes, and/or another addictive drug. The scale for close friend substance use was constructed by dichotomous ‘yes’ or ‘no’ answer. Respondents were asked about history of family drug use. Responses by youth who reported ever having a family member who used drugs were dichotomized as ‘yes’ and ‘no’.

Sexual behavior was measured by the dichotomous (yes/no) question: ‘Have you ever had sexual intercourse?’ This was followed by a question on history of vaginal, anal, and/or oral-genital sex. Response indicating a history of any of these behaviors was coded as 1, while no history was coded as 0.

### Statistical analysis

To examine whether no e-cigarette, e-cigarette use only, and e-cigarette + other substance(s) use was associated with sociodemographic characteristics, a multinomial logistic regression analysis was performed, given the categorical nature of the dependent measure. Adjusted odds ratios with a 95% confidence interval from the multinomial logistic regression are reported. We conducted data analysis using Stata and SPSS, using survey procedures to account for the complex survey design. First, we calculated descriptive statistics (i.e. frequencies and percentages) for substance use, past 30-day substance use, and e-cigarette poly-use status. We further characterized patterns of use by producing frequency counts and percentages for combinations of individual substances used among e-cigarette poly-users. Second, we examined e-cigarette poly-use status by sex and grade, as well as the past-30-day frequency of e-cigarette use by poly-use status. Finally, we used multinomial logistic regression to examine the adjusted relationships between sociodemographic characteristics and e-cigarette poly-use status.

## RESULTS

The total sample consists of 6167 students from public school grades 8 and 11, and year II public vocational students. More than half the sample was female (53.5%) (Table 1). Most had good academic achievement at A or B grade levels (73.6%). Half of the sample (50.6%) had a close friend(s) who drinks alcohol, over one-third (37.1%) had a close friend(s) who smokes cigarettes, and over one in ten (10.8%) have a close friend(s) who uses other addictive drugs. In addition, about one in eight (13.0%) had a close friend(s) who persuaded them to use drugs. One out of seven (14.1%) reported ever having sex, and female students were slightly more sexually-active than their male counterparts (15.3% and 12.8%, respectively).

**Table 1:** The proportions for all three groups: no e-cigarette use, e-cigarette-only use, and e-cigarette use with other addictive substances, by independent variables

*Variables*	*Categories*	*Total*		*E-cigarette status past 30 days*					
				*No use*		*Single use*		*Poly use*	
		*n*	*%*	*n*	*%*	*n*	*%*	*n*	*%*
Academic achievement*	A or B	4538	73.6	4284	94.4	69	1.5	185	4.1
	C or D	1629	26.4	1467	90.0	47	2.9	115	7.1
Close friend alcohol use	No	3046	49.4	2980	97.9	41	1.3	25	0.8
	Yes	3121	50.6	2771	88.8	75	2.4	275	8.8
Close friend cigarette use	No	3882	62.9	3806	98.0	35	0.9	41	1.1
	Yes	2285	37.1	1945	85.2	81	3.5	259	11.3
Close friend other substance use	No	5501	89.2	5211	94.7	97	1.8	193	3.5
	Yes	666	10.8	540	81.0	19	2.9	107	16.1
Close friends persuade to use substance	No	5364	87.0	5120	95.4	84	1.6	160	3.0
	Yes	803	13.0	631	78.6	32	4.0	140	17.4
Sexual experience	No	5295	85.9	5060	95.5	84	1.6	151	2.9
	Yes	872	14.1	691	79.2	32	3.7	149	17.1
Household drug use	No	5150	83.5	4829	93.7	96	1.9	225	4.4
	Yes	1017	16.5	922	90.6	20	2.0	75	7.4
Sex	Male	2869	46.5	2607	90.0	77	2.7	185	6.4
	Female	3298	53.5	3144	95.3	39	1.2	115	3.5
Grade	8	2093	33.9	2008	95.9	32	1.5	53	2.5
	11	2052	33.3	1950	95.0	22	1.1	80	3.9
	Vocational	2022	32.8	1793	88.7	62	3.1	167	8.3

* Academic achievement at A, B, C or D grade level.

Estimates of the prevalence of substance use (Table 2) show that the highest percent had consumed alcohol in the past 30 days, followed by a similar proportion of those smoking e-cigarettes and conventional cigarettes and marijuana. Single drug use (only e-cigarettes) accounted for 2% percent of the total sample. One in twenty (4.9%) smoked e-cigarettes and used other substances in the past 30 days. The combination of e-cigarettes and substance abuse was mixed. However, the frequency of alcohol consumption along with smoking e-cigarettes and tobacco was highest, followed by smoking e-cigarettes and consuming alcohol.

**Table 2:** Prevalence of adolescent substance use, 2019 BBSS (N=6167)

*Variable*		*n*	*%*	*95% Cl*
**Past 30-day substance use**	Alcohol	930	15.1	14.2-16.0
	Cigarette	384	6.2	5.6-6.8
	E-cigarette	416	6.7	6.1-7.4
	Cannabis	122	2.0	1.6-2.4
**E-cigarette poly-use status**	No e-cigarette use	5751	93.3	93.0-93.9
	E-cigarette use only	116	1.9	1.6-2.3
	E-cigarettes + other substance(s)	300	4.9	4.3-5.4
**Substance combinations among e-cigarette poly-users** (n=300)	E-cigarettes + alcohol	101	33.7	28.3-39.3
	E-cigarettes + cannabis	3	1.0	0.8-1.3
	E-cigarettes + tobacco	55	18.3	14.1-23.2
	E-cigarettes + alcohol + tobacco	105	35.0	29.6-40.7
	E-cigarettes + alcohol + cannabis	5	1.7	0.5-3.8
	E-cigarettes + cannabis + tobacco	14	4.7	2.6-7.7
	E-cigarettes + alcohol + cannabis + tobacco	17	5.7	3.3-8.9

**Figure 1:**
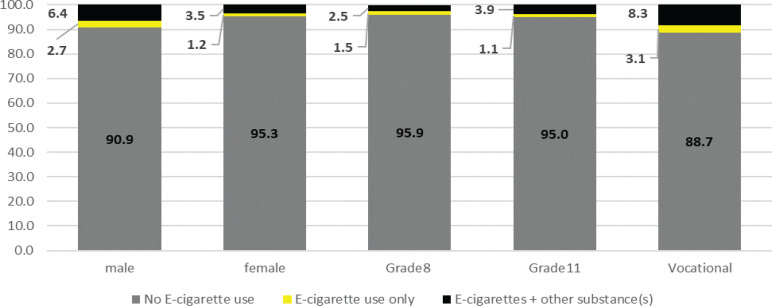
E-cigarette poly-use status by sex and grade

In Figure 1, the proportion of users of all four substances (e-cigarettes + alcohol + cigarettes + marijuana) in the past 30 days increased with age from 2.5%, to 3.9%, to 8.3% among students in grade 8, 11, and vocational year II, respectively. In contrast, the proportion of students using e-cigarettes only was very small (1.1–3.1%). The chi-squared test found a significant, positive association between education level and substance abuse status (Pearson’s χ^2^=105.71, p<0.001). Male students (2.7%) reported use of all four substances more than female students (1.2%). For use of e-cigarettes only, the chi-squared test found a significant positive association between gender and substance abuse status (Pearson’s χ^2^=49.32, p<0.001).

**Figure 2:**
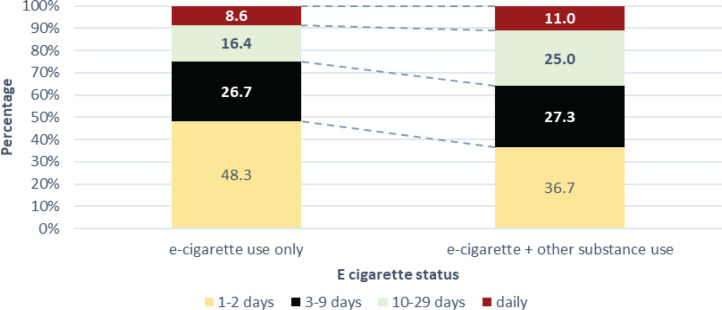
Past 30-day frequency of e-cigarette use by poly-use status, 2019 (N=6167)

The results of the frequency analysis of use of e-cigarettes over the past 30 days are shown in Figure 2. The analysis compared the frequency of e-cigarette use over the past 30 days in the e-cigarettes-only and e-cigarettes + polysubstance use groups. Those who used e-cigarettes only reported slightly higher use in the past 30 days compared to the polysubstance users (48.3% vs 36.7%, respectively). However, this difference was not statistically significant.

Table 3 presents the results of the multinomial logistic regression analysis for the status of e-cigarette-only use and e-cigarette use with other addictive substances in the past 30 days (compared to no e-cigarette use). Those who used e-cigarettes only were male students (OR=1.48), year II vocational students (OR=1.41), had academic achievement below grades A and B (OR=1.54), had ever had sex (OR=2.85), had close friends persuade them to use drugs (OR=2.51), and had close friends who smoked (OR=2.92). Those who used e-cigarettes concurrently with other addictive substances (compared to not using e-cigarettes) were male (OR=1.51), year II vocational students (OR=1.54), 11th graders (OR=1.32), had academic achievement below grades A and B (OR=1.67), had ever had sex (OR=3.70), had close friends persuade them to use drugs (OR=2.88), had close friends who smoked (OR=3.44), and whose best friend drinks alcohol (OR=3.26).

**Table 3:** Multinomial logistic regression for e-cigarette poly-use status, 2019 BBSS (N=6167)

*Variables*	*Categories*	*E-cigarette status past 30 days*						
		*No-use^a^*	*E-cigarette single use*			*E-cigarette poly use*		
			*OR*	*95% CI*		*OR*	*95% CI*	
				*Lower*	*Upper*		*Lower*	*Upper*
Academic achievement (Ref: A + B)	C + D	1	**1.54**	1.37	1.78	**1.67**	1.51	1.87
Close friend alcohol use (Ref: No)	Yes	1	1.24	0.76	2.03	**3.26**	2.04	5.21
Close friend cigarette use (Ref: No)	Yes	1	**2.92**	1.76	4.85	**3.44**	2.34	5.07
Close friend another drug use Ref: No)	Yes	1	1.54	0.88	2.71	1.11	0.82	1.51
Close friends persuade (Ref: No)	Yes	1	**2.51**	3.13	8.01	**2.88**	2.17	3.82
Sexual experience (Ref: No)	Yes	1	**2.85**	3.70	9.24	**3.70**	2.82	4.86
Household drug use (Ref: No)	Yes	1	1.02	0.61	1.68	1.34	0.99	1.80
Gender (Ref: Female)	Male	1	**1.48**	1.32	1.73	**1.51**	1.39	1.67
Grade (Ref: Grade 8)	11	1	0.65	0.40	1.07	**1.32**	1.02	1.71
	Vocational	1	**1.41**	1.25	1.68	**1.54**	1.37	1.79

a Reference category: No e-cigarette use (n=5751). Significant findings (p<0.05) highlighted in bold.

## DISCUSSION

The usage rate of e-cigarettes in Thailand is increasing rapidly. Marketing strategies are aimed at young people who are impressionable, easy-going, believe that e-cigarettes can help them quit smoking conventional cigarettes, and believe that vaping is a healthier way to consume nicotine^21-23^. However, there are no reliable data which show that use of e-cigarettes helps combat addiction to conventional cigarettes.

The Thai government has banned the import of e-cigarettes since 2014. The Thai Consumer Protection Board issued an order prohibiting the sale or service of e-cigarettes in 2015, and the 2017 Tobacco Products Control Act classified e-cigarettes in the same category as conventional cigarettes. Nevertheless, e-cigarettes are being marketed to Thais through the Internet and various underground channels at/around markets, entertainment establishments, and other places where Thai youth congregate^24^. Most e-cigarette users in Thailand use them along with conventional cigarettes (dual usage) rather than using e-cigarettes to completely replace conventional cigarettes^12,25^. Some Thai users of e-cigarettes lack knowledge and understanding about the products they use^2^.

This study found that the majority of adolescents in this sample were not current users of e-cigarettes. However, polysubstance use was widespread: almost all adolescents using e-cigarettes also used other substances. However, very little research has been done on the combined use of e-cigarettes with other substances. Since the patterns of adolescent substance use change over time – sometimes relatively quickly – precise epidemiological patterns need to be studied to inform an effective public health response. In addition to studying vaping behavior in combination with smoking conventional cigarettes, this study also adds to the knowledge base by expanding the scope of e-cigarette research to examine the use of alcohol and cannabis concurrently with e-cigarette use.

The prevalence of e-cigarettes increased with increasing grade of the students, i.e. from the 8th and 11th grades of public school, to the 2nd year of public vocational school. This may reflect age bias and the pattern of smoking initiation among Thai youth in general. The finding is consistent with data from studies in the US which found that consuming e-cigarettes led to earlier age of addiction^26,6^. Prevalence of e-cigarette use in the US increased rapidly from 2011 to 2015 (from 1.5% to 16.0%), then showed a slight decline between 2015 and 2017. However, the trend in use increased again between 2017 and 2019 (from 11.7% to 27.5%). Studies in the US found that e-cigarettes may replace traditional tobacco products among some youth^27,28^.

In this study of Thai youth, a small proportion reported using e-cigarettes only, and that proportion increased from the 8th to 11th grade, and to year II vocational students. As noted above, most e-cigarette users also used another substance in the past 30 days, and 6% reported drinking alcohol, smoking cigarettes, smoking marijuana, and e-cigarette use, though not necessarily at one sitting. Previous studies have found similar levels of polysubstance use. One study found that users of two substances (compared with those who used e-cigarettes or cigarettes only) had a significantly higher risk of polysubstance use behaviors^17^. The consistency of these emerging findings underscores the need to expand the focus of efforts to protect adolescents from progressing from single substance to polysubstance abuse.

The results of the study on e-cigarette use frequency – not just prevalence – provide further insight into behavior patterns. Although no statistically significant association was found between the frequency of e-cigarette smoking and some key variables, the frequency of e-cigarette use is increasing among vocational school students. It is possible that, among those students, a greater proportion of users of e-cigarettes will have developed a dependency on nicotine. If so, more research is needed to determine if this is a consequence of the use of e-cigarettes or other tobacco products. This study also found a significant link between polysubstance use and frequency of e-cigarette use. We found a higher proportion of daily and moderate use of e-cigarettes among students with polysubstance use behavior compared to users of e-cigarettes only. Thus, students who use multiple, addictive substances may experience both a wider range and greater intensity of exposure to drugs, and that could result in more harm^6^.

The regression model that was applied in this study identified several sociological factors associated with polysubstance use with e-cigarettes. Being a 2nd year vocational student with low academic achievement, having ever had sex, having a close friend persuade them to use drugs, and having close friends who smoke and drink alcohol were associated with significantly higher use of e-cigarettes. These patterns are consistent with the results of a cohort study of students using e-cigarettes only. The point from this analysis is that there may be sociodemographic factors underlying the use of various substances^17,29^. In addition, the results of this study provide information to guide substance abuse prevention programs among students and adolescents.

The findings have several important implications. First, because of the high prevalence of users of e-cigarettes, the results indicate that prevention interventions among school-age youth must address polysubstance abuse simultaneously. In implementing these measures, it is advisable to take a holistic approach to the substance abuse mentioned above. Second, those measures need to be coupled with interventions that increase in intensity as the education level increases. Therefore, the importance of screening for repeated use of e-cigarettes with various substances should be emphasized^18^. To facilitate the expansion of screening for the use of e-cigarettes for substance abuse, it is also recommend that outreach efforts be made in a variety of contexts, including in the community and in the workplace^30^. This may include conducting supplementary activities within the school, such as scout rallies, academic camps, sports competition, academic counseling sessions, and social club activities. Interventions should be targeted at places in communities frequented by teenagers, such as recreational programs and charities organized by temples, youth councils, or local government agencies^4^. However, it is recognized that the effectiveness of screening and prevention activities in places other than the school must be established through a research process with additional intervention measures^31^. Finally, it is believed that positive screening for the use of e-cigarettes should be encouraged to include follow-up testing for the presence of other addictive substances^32,33^. There are validated screening tools for this. However, because of the negative social stigma about youth and alcohol consumption, the prevention education should initially focus on e-cigarette use and e-cigarettes^34^. This approach may serve as a starting point for evaluating use of other addictive substances.

### Strengths and limitations

This study has several strengths. These include the use of a large dataset for students in urban areas. This allows for effective estimates of adolescent substance use and analysis based on social characteristics. That information may guide preventive and corrective measures against drugs in the target population, as well as provide a basis for increasing awareness about e-cigarettes due to word-of-mouth referrals from fellow smokers. The study’s findings, however, should be considered with caution based on a number of potential limitations. First, the questionnaire was self-administered. Thus, the level of substance use is likely under-reported^35^. Second, a previous study found that e-cigarette use was associated with higher income. However, this study did not include measures of economic status of the youth/household. That limits the ability to draw conclusions about the findings. Future studies with a larger sample size should include indicators of income or economic status of the household. Third, the study was unable to ascertain the duration of polysubstance use. It is possible that the risk of using two or more substances concurrently will be different compared to using two or more substances at different times. Similarly, the study did not analyze the age of onset of substance use. It is possible that new users of e-cigarettes may be new users of other addictive substances^36^. Alternatively, youth may initiate use of two or more substances at the same time^12^. Conversely, the consumption of e-cigarettes may occur after a period of use of another addictive substance^26^. Future research should assess both the initiation and duration of polysubstance use to address these questions, as it will clarify the pattern and order of substance abuse among adolescents. Fourth, this study was an analysis of secondary data. Variable selection was limited by the source study design. In particular, the BBSS survey did not look at specific characteristics of e-cigarettes that may be involved, such as the type of device, use of flavoring, or the composition of nicotine and/or tetrahydrocannabinol as the main component of e-cigarettes, and their primary function to generate aerosol vapors. E-cigarettes contain many chemicals that may produce a different reaction in different people. Studies on the health risks of inhalation are limited. In addition, previous studies have found that psychological factors (e.g. emotional state, personal problems) are related to the use of e-cigarettes^24^. In fact, these factors are commonly associated with youth substance abuse generally. Therefore, future adolescent substance use studies should collect information about the characteristics of e-cigarettes, as well as psychological and personality traits to expand the understanding of the risk factors associated with the use of e-cigarettes and polysubstance use. This study addresses polysubstance use with regard to e-cigarette consumption and onset of alcohol use. The persistent public health problem is likely to be related to alcohol misuse in the adolescent population. Thus, the dual use of e-cigarettes and alcohol^15^ (similar to that of smoking and alcohol consumption) needs to be investigated further. Due to the design of this study, no information on alcohol misuse was available. Therefore, a significant volume of research is still needed on alcohol misuse to better understand the cross-fading potential for simultaneous electronic cigarette and alcohol abuse.

## CONCLUSIONS

This study expands knowledge about the use of e-cigarettes among urban Thai adolescents by examining the use of e-cigarettes with use of alcohol, marijuana, and rolled tobacco. This study found that the majority of adolescents who use e-cigarettes are polysubstance users. In addition, several sociodemographic factors were analyzed that were associated with an increased likelihood of polysubstance use and e-cigarette use. Based on the findings of this study, preventive measures are recommended, including a focus on polysubstance use, and repeated screening to detect the onset of e-cigarette use with increasing age. In prevention education, the emphasis should be first placed on the use of e-cigarettes as a starting point for broader action on substance abuse prevention and treatment. The target population should be prioritized for level of risk of addiction. This study adds to the body of knowledge about e-cigarette use among adolescents by exploring the concurrent use of e-cigarettes with alcohol, marijuana and other tobacco products. The analysis shows that teenagers are not using e-cigarettes only. The results of the analysis identified several sociodemographic factors associated with an increased likelihood of polysubstance use in conjunction with e-cigarette use. In response to the study results, preventive measures are proposed to deal with polysubstance use combined with repeated screening to detect relapse. A starting point for prevention education is to avoid methods which stigmatize use of e-cigarettes, and there should be careful targeting of the priority risk groups.

## Data Availability

The data supporting this research are available from the authors on reasonable request.
